# Letter from the Editor in Chief

**DOI:** 10.19102/icrm.2020.110501

**Published:** 2020-05-15

**Authors:** Moussa Mansour


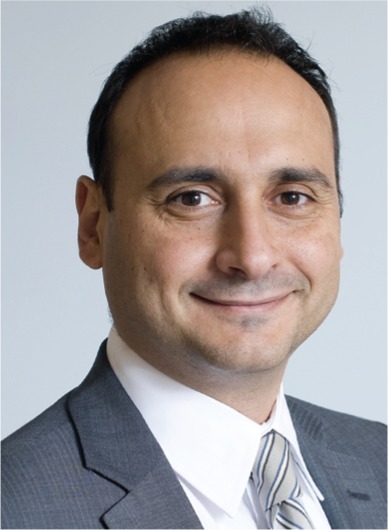


Dear Readers,

The lives of both electrophysiologists and their patients have been upended by the spread of COVID-19 and subsequent lockdown of parts of the country, including the cancellation of elective medical procedures. While some organizations like the Heart Rhythm Society,^1^ the Latin American Heart Rhythm Society,^2^ and the Italian Association of Hospital Cardiologists^3^ have released guidance for determining how to proceed with cardiac procedures that cannot be delayed, the anticipated impact of this widespread cessation is of significant concern for two reasons. First, the delay may cause patients initially scheduled for elective procedures to progress to needing emergency ones and, second, when things finally do open up again, the workload of both electrophysiologists and other health care practitioners is expected to multiply as we seek to play catch-up, thus potentially further delaying or complicating procedures that have been scheduled for later this year in a kind of ripple effect.

At this time, the federal government has released a phased approach to reopening,^4^ which includes the reinstation of elective medical procedures as part of phase I. However, these guidelines are only such and it is likely, as we are already seeing in some cases, that each state will adopt its own unique approach to reopening, relying on data and recommendations from a variety of specialty groups including health care practitioners.

Knowing the virus will be present in our communities for many months to come, we will have to find ways to begin performing electrophysiology procedures again while simultaneously introducing new protocols to better protect our patients and staff. To this end, I recently contacted colleagues from countries affected by COVID-19 before the United States who are currently on their way to recovery. From our discussion, it seems that most are relying on large-scale testing for the virus of both patients and staff. The model largely being adopted consists of testing the patient before their elective procedure and proceeding with the operation if the test is negative. Despite that this model requires significant resources, it appears that it is the most successful approach available so far and will probably be adopted in most hospitals around the world for the foreseeable future.

Meanwhile, nonprocedural clinical care has been less affected by COVID-19 because of past advances in telemedicine, although the virus has perhaps prompted a much speedier adoption of the technology^5^ that has not been without its challenges. Still, since the crisis began, most of us have been able to conduct virtual visits and provide effective care in a way that many did not think was possible before the outbreak and it is likely that this will remain in effect to some degree.

I want to take this opportunity during Nurses’ Appreciation Week to recognize and thank all nurses for their dedication to their patients. Nurses have consistently been on the front lines of the COVID-19 fight and their efforts have been nothing short of heroic.

Sincerely,


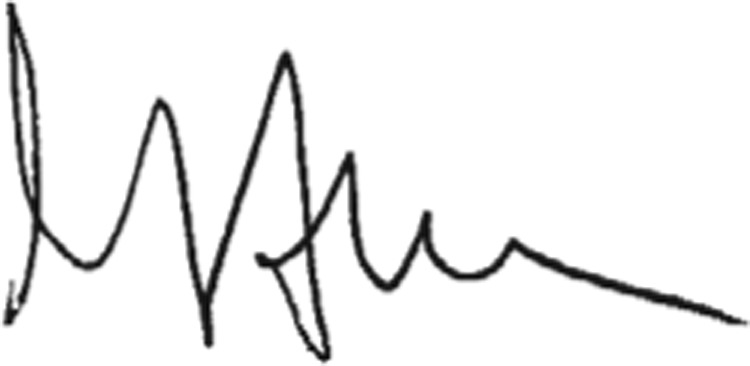


Moussa Mansour, MD, FHRS, FACC

Editor in Chief

The Journal of Innovations in Cardiac Rhythm Management

MMansour@InnovationsInCRM.com

Director, Atrial Fibrillation Program

Jeremy Ruskin and Dan Starks Endowed Chair in Cardiology

Massachusetts General Hospital

Boston, MA 02114
